# Analysis of variant-pairing tendencies in lenticular martensite microstructures based on rank-1 connection

**DOI:** 10.1038/s41598-021-93514-z

**Published:** 2021-07-22

**Authors:** Yuri Shinohara, Satomu Akabane, Tomonari Inamura

**Affiliations:** 1 Laboratory for Materials and Structures, Tokyo Institute of Technology, 4259-J3-22, Nagatsuta, Midori-ku, Yokohama, 226-8503 Japan; 2 Laboratory for Future Interdisciplinary Research of Science and Technology, Tokyo Institute of Technology, 4259-J3-22, Nagatsuta, Midori-ku, Yokohama, 226-8503 Japan; 3 Tokyo Institute of Technology, 4259-J3-22, Nagatsuta, Midori-ku, Yokohama, 226-8503 Japan

**Keywords:** Structural materials, Theory and computation

## Abstract

Herein, variant-pairing tendencies of lenticular martensite in an Fe–30Ni–0.3C (wt%) alloy are analyzed based on rank-1 connection at martensite/martensite junction planes (JPs) to facilitate the understanding of martensite microstructure. The degree of incompatibility (*θ*) at the JPs successfully explained their observed frequency; in the actual microstructure, variant pairs with a small *θ* form preferentially. The experimentally obtained JPs were consistent with theoretical ones. To the best of our knowledge, this is the first study to confirm the validity of variant-pair crystallography in steel based on rank-1 connection, both theoretically and experimentally. Diamond, composite-spear, and composite-kink clusters are considered. The cumulative *θ* at the JPs can suppress diamond cluster formation because it exceeds the *θ* of a single variant pair, and the diamond cluster is not observed experimentally. However, *θ* at the JPs cancel out in composite-spear (CS) and composite-kink (CK) clusters, but CK clusters are rarely observed experimentally, while a few CS clusters are observed. This demonstrates the analytical limitations of 2D approaches used to evaluate the frequency of variant pairs and clusters. These two variant clusters have a narrow window of 2D observation because the orientation relationships between JPs and intersection lines between two habit planes affect the areas of JPs.

## Introduction

**Figure 1:**
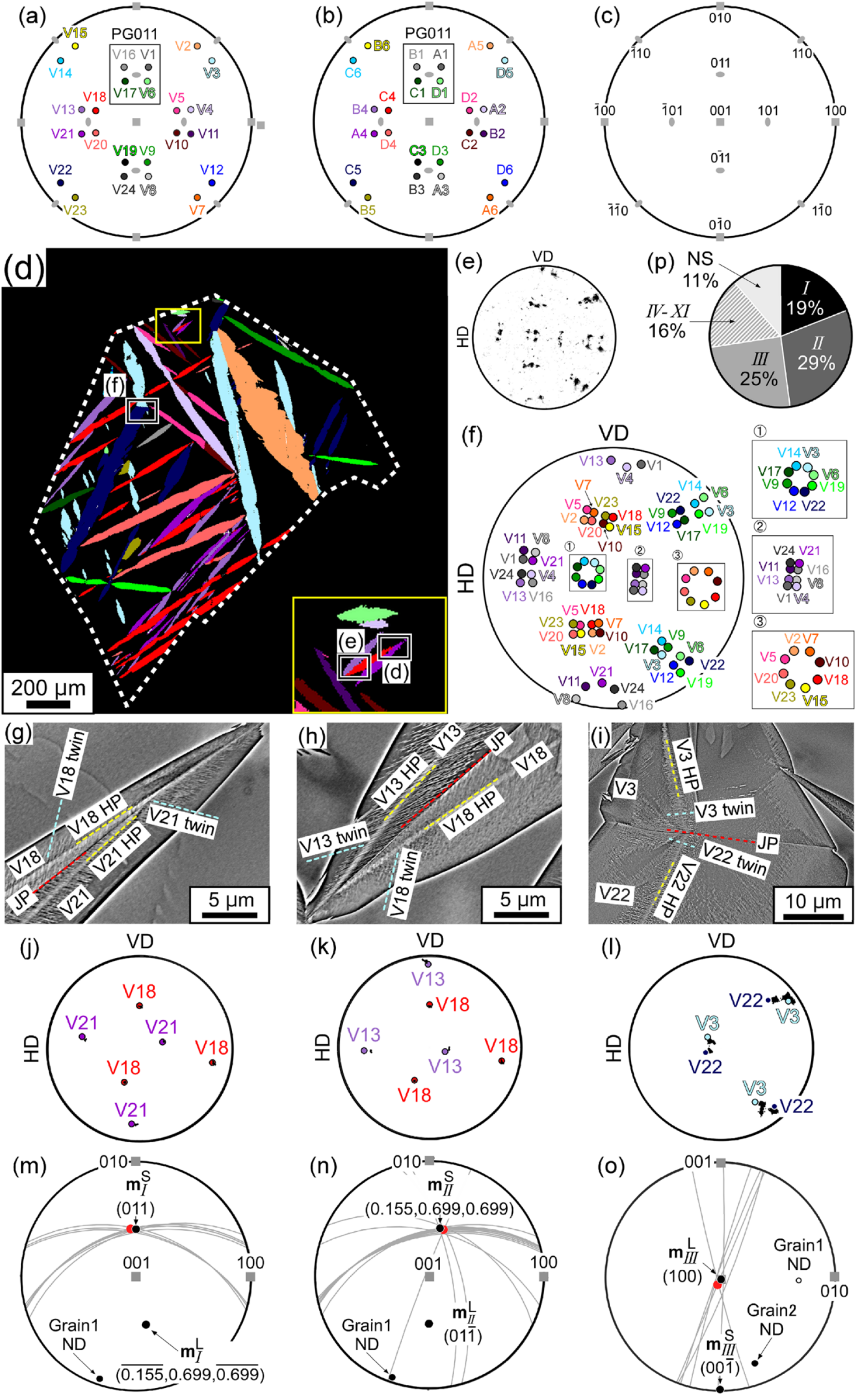
Stereographic projections showing the **p**_*k*_ of the 24 variants with respect to austenite lattice in (**a**) the K–S notation^1^ and (**b**) the notation presented by Okamoto^2^. (**c**) A key diagram of the 001 and 110 poles. (**d**) Variant map of Grain 1 in the specimen cooled at 231 K. The prior austenite grain boundary is depicted by a white dashed line. The inset is an enlarged view of the region framed in yellow. (**e**) 100_M_ PF of martensite variants in Grain 1. (**f**) Positions of the 100_M_ poles of 24 variants when the martensite satisfies the K–S OR. Typical morphology of (**g**) type *I*, (**h**) type *II,* and (**i**) type *III* variant pairs identified in (**d**). The theoretical traces of twins, HPs, and JPs are depicted by light-blue, yellow, and red dashed lines, respectively. (**j**)–(**l**) Corresponding PFs of the variant pairs in (**g**)–(**i**). The positions of the theoretical 100_ M_ poles (K–S OR) are also shown. Determination of JP orientations by single trace analysis for (**m**) type *I*, (**n**) type *II*, and (**o**) type *III* variant pairs. The theoretical and average orientations of the JPs (determined experimentally) are shown in red and black, respectively. The specific indices are presented in Table S2. Poles of ${\mathbf{m}}_{{{I}}}^{{{\text{L}}}}$ and ${\mathbf{m}}_{{{{II}}}}^{{{\text{L}}}}$ are symmetry converted about the origin. (**p**) Observed frequencies of the variant pairs in 7 prior austenite grains.

Martensite microstructure is an essential feature of high-strength steel. When martensitic transformation occurs by cooling in the absence of external stress, biases in adjacent variants are observed. These biases have been studied from the perspective of characteristic martensite morphologies (lath, butterfly, lenticular, and thin-plate)^1–10^. As for α′ martensite (body-centered cubic or body-centered tetragonal), Morito et al*.* proposed 24 Kurdjumov–Sachs (K–S) equivalent variants formed in a single austenite (face-centered cubic) grain^1^. The typically preferred variants adjacent to V1 are V4 and V2 for lath, and V16 for butterfly, lenticular, and thin-plate martensite^1,3,5,10^. The V1 variant corresponds to the A1 variant in the notation adopted by Okamoto to describe thin-plate martensite (see Fig. 1a–c and Supplemental Fig. S2)^2^. We employ the K–S notation in principle. Similarly, the typically preferred variants adjacent to A1 are A2 and A5 for lath, and B1 for lenticular and thin-plate martensite.

The adjacent-variant bias in lenticular and thin-plate martensite is partially explained by self-accommodation, similar to shape memory alloys (SMAs)^2,3,10–14^. Lattice invariant deformation (LID) introduces complete twinning in thin-plate martensite^15^, forming the midrib of lenticular martensite^16^. The crystallography of an isolated martensite plate is determined by the invariant plane (IP) condition at the habit plane (HP) according to the phenomenological theory of martensite crystallography (PTMC)^17–22^. Two adjacent variants, constituting a variant pair, form to accommodate the shape strain experienced by an isolated pre-existing plate^13^. However, variant-pairing tendencies observed in practice cannot be fully explained by self-accommodation. For example, assemblies of four unique variant plates with near-parallel HPs are rarely observed, although the clustering of these variants is common^2,23^; four variants can accommodate shape strain more effectively than two variants^2^. Groups and assemblies of these variants are called plate groups (PGs) and variant clusters, respectively^2^.

To elucidate the martensite microstructure, we focused on the incompatibilities at martensite/martensite junction planes (JPs). Recently, the martensite microstructures of various materials, such as SMAs and steel, were analyzed in terms of these incompatibilities^24–36^. An important indicator of incompatibility at the JP, namely, degree of incompatibility, is derived below.

The condition for geometric compatibility of two domains with deformation gradients **F** and **G**, i.e., the condition for maintaining continuity of deformation across an interface with normal **n,** is as follows:

1 $${\mathbf{F}} - {\mathbf{G}} = {\mathbf{a}} \otimes {\mathbf{n}},$$

where **a** is a vector that indicates the discontinuity of deformation gradients^37,38^; this is “rank-1 connection” or “kinematic compatibility condition.” The list of notations used in this paper is given in Supplemental Table S1.

For variant pairs, based on the geometrically nonlinear theory of martensitic transformation, Eq. (1) can be rewritten as^38^

2 $$\mathbf{Q}_{{{l/k}}} {\mathbf{P}}_{{l}} - {\mathbf{P}}_{{k}} {{ = }}{\mathbf{b}}_{{{l/k}}} \otimes {\mathbf{m}}_{{{l/k}}} .$$

In Eq. (2), when the variants *k* and *l* with the shape strains **P**_*k*_ and **P**_*l*_, respectively, meet the IP condition, the rigid rotation **Q**_*l/k*_ is required for variant *l* to connect to variant *k* continuously at the JP (with plane normal $\mathbf{m}_{l/k}$ in the austenite coordinate system before transformation). Generally, **Q**_*l/k*_ and its magnitude *θ*_*l/k*_ differ for each variant pair. Only if **Q**_*l/k*_ = **I** (*θ*_*l/k*_ = 0°), both variants *k* and *l* meet the IP condition and variant *l* connects to variant *k* at a JP without any rotation. Thus, *θ*_*l/k*_ can be regarded as the degree of incompatibility at the JP, and thus, of stored energy of the variant plates that impairs variant-pair formation^34^.

Some studies have investigated the martensite microstructure of steel based on the incompatibilities at the JPs. Bhattacharya reported the possibility of a wedge microstructure in thin-plate martensite in terms of the incompatibilities at the JPs^30^. Okamoto et al*.* calculated the indices of JPs for several variant pairs using the plane of intersection of the strain ellipsoid^2^. Koumatos et al. investigated the features of JPs based on rank-1 connection theoretically^39–41^. Kundin performed a phase-field simulation of lenticular martensite microstructure from the perspective of microstructure evolution^42^. Miyamoto et al*.* analyzed the microstructure of lenticular martensite based on the degree of incompatibility at the midrib. They reported that the variant pairs that form preferentially have a small *θ*_*l/k*_^28^. A similar tendency was observed in butterfly martensite^35^.

However, the crystallography of variant pairs derived in terms of rank-1 connection (described by Eq. (2)) and the corresponding experimental results have not been compared in detail. In particular, the degree of incompatibility, theoretical morphology, experimental morphology, and observed frequency of variant pairs or clusters have not been systematically correlated.

Herein, the variant-pairing tendency of lenticular martensite in an Fe-30Ni-0.3C alloy was analyzed based on rank-1 connection. First, the degree of incompatibility at the JPs of all possible variant pairs was evaluated. Then, the validity of theoretical crystallography of the variant pairs was investigated, and the effect of *θ*_*l/k*_ on the frequency of variant pairs was observed. Further, the formation possibility of various types of variant clusters was explored. We believe this study will cultivate an understanding of the martensite microstructure because the martensite nucleation process was considered to correlate with the crystallography of JPs directly. Further, we discuss the analytical limitations of 2D approaches used to evaluate the frequency of variant pairs and clusters. This study will provide a vital future direction for 3D martensite microstructure analysis.

## Results

### Crystallography of martensite plates

The lattice parameters of austenite (*a* = 358.71 ± 0.02 pm) and martensite (*a*_M_ = *c*_M_ = 286.24 ± 0.13 pm) were obtained from a specimen cooled at 77 K; however, a specimen cooled at 231 K would have served equally well because all specimens were confirmed to retain austenite. Lattice parameters, vectors, and matrices without and with the subscript M refer to austenite and martensite, respectively. A {112}_M_ twin with a width of ~ 10 nm is introduced as LID at the midrib, and the volume fraction of the minor twin is ~ 33% (see Supplemental Fig. S1).

### Theoretical evaluation of the IP condition and crystallography of variant pairs

We consider that thin-plate pairs are formed at the earliest stage of martensitic transformation, followed by the occurrence of lenticular growth, because the thickening of lenticular martensite is an extremely slower process than lengthwise growth^43^. This indicates that the shape strain at the midribs represents that of the entire variant plates (see Sect. 5.2). The theoretical volume fraction of the minor twin derived using the PTMC is 37%, which is consistent with the experimental value obtained by TEM (i.e., 33%).

The shape-change vector **d**_*k*_ and the HP normal **p**_*k*_ for the 24 possible variants (*k* = 1–24) were calculated using Eq. (3) (see Supplemental Fig. S2). The variants *k* and *l* are labeled using the K–S notation adopted by Morito^2^. Additionally, we employ the notation adopted by Okamoto^2^ as necessary, because this notation is useful in considering variants belonging to the same PG. Stereographic projections showing the **p**_*k*_ of the 24 variants with respect to the austenite lattice in the K–S notation^1^ and the notation adopted by Okamoto^2^ are shown in Fig. 1a–c. The variants associated with the same number (*n* = 1–6) belong to the same PG and their HPs are located around a single 110 pole. For example, the HPs of A1, B1, C1, and D1 are packed around the 011 pole. There are six PGs, as defined in Supplemental Fig. S3. **d** and **p** for V1 (**d**_V1_ and **p**_V1_) are calculated as [0.045, $\overline{{{{0}}{{.158}}}}$, 0.158] and ($\overline{{{{0}}{{.175}}}}$, $\overline{{{{0}}{{.769}}}}$, $\overline{{{{0}}{{.615}}}}$), respectively, and the other 23 variants have equivalent crystallographic indices.

The crystallography of JPs between variants was evaluated based on rank-1 connection (Eq. (2)). There are 11 solution groups, and each solution group has two *θ*_*l/k*_ values (smaller and larger), rotation axes, and $\mathbf{m}_{\textit {l} /k}$ values. The solution groups are labeled with Roman numerals in increasing order of *θ*_*l/k*_, and the *θ*_*l/k*_ and JP values are identified according to the solution group number and magnitude of *θ*_*l/k*_. For example, ${\mathbf{m}}_{{{I}}}^{{{\text{S}}}}$ and ${\mathbf{m}}_{{{I}}}^{{{\text{L}}}}$ indicate the JPs of variant pairs belonging to the solution group *I* with smaller and larger *θ*_*l/k*_ ($\theta _{{{I}}}^{{{\text{S}}}}$ and $\theta _{{{I}}}^{{{\text{L}}}}$), respectively, i.e., (0, 0.707, 0.707) and ($\overline{{{{0}}{{.155}}}}$, 0.699, $\overline{{{{0}}{{.699}}}}$), respectively, in the case of the V1/V17 variant pair. Note that ${\mathbf{m}}_{{{I}}}^{{{\text{S}}}}$ and ${\mathbf{m}}_{{{I}}}^{{{\text{L}}}}$ do not indicate the plane normal but the plane itself. Table 1 lists the *θ*_*l/k*_ values, rotation axes, and JPs when the variant V1 is adjacent to the other 23 variants. JPs with a normal that is not perpendicular to the intersection lines of their two associated HPs (i.e., the intersection line of two HPs does not lie on the associated JP) are highlighted in Table 1 and discussed in Sect. 3. The solution groups of all 552 possible variant pairs are listed in Fig. S3.

**Table 1:** Solution groups based on rank-1 connection between V1 and the 23 other variants (variant notation presented by Okamoto is given in parentheses). Rotation axis and **m**_*l/k*_ are presented in the austenite coordinate system*.* NS means no solution. The JPs with normals that are not perpendicular to the intersection lines of their two associated HPs are in bold.

Variant		Type of solution	*θ*_*l/k*_ (smaller) (larger)	Rotation axis	**m**_*l/k*_
*k*	*l*				
V1 (A1)	V16 (B1)	*III*	4.51	[0, 0.708, 0.706]	(**00**$\boldsymbol{\bar{1}}$)
			4.95	[0, $\overline{{{{0}}{{.625}}}}$, 0.781]	(100)
	V17 (C1)	*I*	5.94 × 10^–3^	[$\overline{{0.529}}$, 0.600, $\overline{{0.600}}$]	(0, 0.707, 0.707)
			25.2	[$\overline{{0.980}}$, $\overline{{0.139}}$, 0.139]	($\boldsymbol{\overline{0.155}}$, **0.699**, $\boldsymbol{\overline{0.699}}$)
	V6 (D1)	*II*	0.547	[$\overline{{0.006}}$, 0.707, 0.707]	(0.155, 0.699, 0.699)*
			25.3	[$\overline{{0.984}}$, 0.124, 0.124]	(0, 0.707, $\overline{{0.707}}$)
	V4 (A2)	*VI*	8.04	[$\overline{{0.631}},\;\overline{{0.631}},\;\overline{{0.451}}$]	($\boldsymbol{\overline{0.687}},\;\boldsymbol{\overline{0.687}},\;\boldsymbol{\overline{0.237}}$)
			15.5	[$\overline{{0.479}},\;\overline{{0.479}}$, 0.735]	(0.707, $\overline{{0.707}}$, 0)
	V11 (B2)	*VIII*	11.4	[$\overline{{0.627}}$, $\overline{{0.395}}$, 0.672]	($\overline{{0.687}}$, 0.687, $\overline{{0.237}}$)
			11.9	[$\overline{{0.392}}$, $\overline{{0.704}}$, $\overline{{0.591}}$]	(**0.707**, **0.707**, **0**)
	V24 (B3)	*X*	12.8	[$\overline{{0.962}}$, $\overline{{0.274}}$, 0]	(**100**)
			12.8	[$\overline{{0.975}}$, 0.221, 0]	(00$\bar{1}$)
	V19 (C3)	*XI*	12.8	[0.970, $\overline{{0.242}}$, 0.027]	(0.155, 0.699, 0.699)
			12.9	[$\overline{{0.962}}$, 0.000, 0.273]	(**0**, **0.707**, $\boldsymbol{\overline{0.707}}$)
	V9 (D3)	*XI*	12.8	[$\overline{{0.970}}$, 0.027, 0.242]	($\overline{{0.155}}$, 0.699, $\overline{{0.699}}$)
			12.9	[0.962, 0.273, $\overline{{0.000}}$]	(**0**, **0.707**, **0.707**)
	V21 (A4)	*IV*	7.07	[$\overline{{0.584}}$, 0.584, $\overline{{0.564}}$]	(0.707, 0.707, 0)
			15.4	[$\overline{{0.524}}$, 0.524, 0.672]	($\boldsymbol{\overline{0.687}}$, **0.687**, $\boldsymbol{\overline{0.237}}$)
	V13 (B4)	*VIII*	11.4	[$\overline{{0.395}}$, 0.627, $\overline{{0.672}}$]	($\overline{{0.687}}$, $\overline{{0.687}}$, $\overline{{0.237}}$)
			11.9	[$\overline{{0.704}}$, 0.392, 0.591]	(**0.707**, $\boldsymbol{\overline{0.707}} ,~$ **0**)
	V2 (A5)	*V*	7.80	[$\overline{{0.523}}$, $\overline{{0.673}}$, $\overline{{0.523}}$]	($\boldsymbol{\overline{0.707}}$, **0**, $\boldsymbol{\overline{0.707}}$)
			8.36	[$\overline{{0.572}}$, 0.588, $\overline{{0.572}}$]	($\overline{{0.707}}$, 0, 0.707)
	V23 (B5)	*VII*	10.7	[$\overline{{0.703}}$, $\overline{{0.593}}$, $\overline{{0.392}}$]	(**0.873**, **0**, **0.487**)
			11.1	[$\overline{{0.753}}$, 0.507, $\overline{{0.420}}$]	(0.487, 0, $\overline{{0.873}}$)
	V7 (A6)	*IX*	12.3	[$\overline{{0.631}}$, 0.453, 0.631]	($\boldsymbol{\overline{0.707}}$, **0**, **0.707**)
			12.4	[$\overline{{0.655}}$, $\overline{{0.375}}$, 0.655]	($\overline{{0.707}}$, 0, $\overline{{0.707}}$)
	V15 (B6)	*VII*	10.7	[$\overline{{0.392}}$, 0.593, 0.703]	(**0.487**, **0**, $\boldsymbol{\overline{0.873}}$)
			11.1	[$\overline{{0.420}}$, $\overline{{0.507}}$, 0.753]	(0.873, 0, 0.487)
	V3, V5, V8, V10, V12, V14, V18, V20, V22 (D5, D2, A3, C2, D6, C6, C4, D4, C5)	NS	–	–	–

*Intersection line of the HPs and the normal of the JP has a deviation of 1.2°.

### Comparison of theory with experiment

The theoretical and experimental crystallographic features of type *I*–*III* variant pairs were compared. Plastic deformation occurs predominantly in the austenite surrounding lenticular martensite, resulting in a gradual change in the austenite orientation^44,45^. Thus, the JPs in the specimen cooled at 231 K were analyzed to minimize the effect of any orientation changes. The normal direction (ND) of prior austenite used for analysis is shown in Supplemental Fig. S4. First, a variant map of Grain 1 of austenite was constructed. The orientation spread of austenite is ~ 5°, and the identification of the 24 variants is described below.

The variant map in Fig. 1d was constructed to analyze the typical morphology of type *I*–*III* variant pairs. The 100_ M_ pole figure (PF) constructed from the experimental data obtained by EBSD is shown in Fig. 1e, which is consistent with the simulated positions of the Kurdjumov–Sachs orientation relationship (K–S OR; (111)//(011)_M_, [$\bar{1}$01]//[$\bar{1}\bar{1}$1]_M_) (Fig. 1f). The experimental data are also consistent with the simulated positions calculated from the IP condition, as shown in Supplemental Fig. S5(a). These approaches are valid because midribs consist of thin-plate martensite^16^, whose crystallographic features are well-explained by the IP condition^46^, and lenticular martensite demonstrates a K–S OR and Greninger–Troiano orientation relationship (G–T OR; (111) 1° from (011)_M_, [$\bar{1}$01] 2.5° from [$\bar{1}\bar{1}$1]_M_)^47^. In the case of K–S OR, the minimum misorientation between the reference variant (V1) and every other variant is more than 10°; therefore, all 24 variants are identified because this value is greater than the orientation spread of austenite. The notation proposed by Okamoto is under the IP condition, and the misorientation observed in 22 of the reference variant (A1)/variant pairs exceeds 10°, whereas that observed in one reference variant/variant pair is 3°. The variant combinations with small misorientations are A1/A2, B1/B4, C1/C6, D1/D5, B2/A3, C2/A6, D2/A5, B3/A4, C3/C5, D3/D6, C4/B6, and D4/B5. This result indicates that these variants cannot be distinguished solely using the crystal orientation information, and trace analysis of the HPs is additionally required. The traces of the 24 HPs are shown in Supplemental Fig. S5(b), and the difference in the trace angles of two variants with small misorientations is sufficient for variant identification; the minimum trace angle difference is 14°, which exceeds the orientation spread of austenite. Figure 1g–i show BSE images of type *I*–*III* variant pairs observed in the areas framed in Fig. 1d (see Supplemental Fig. S3). Their corresponding 100_ M_ PFs are shown in Fig. 1j–l, reflecting the theoretical 100_ M_ poles (K–S OR) as well. The theoretical traces of the twins, HPs, and JPs depicted by light-blue, yellow, and red dashed lines are consistent with those observed. As shown in Table 1, each solution group has two *θ*_*l/k*_ values. JPs with $\theta _{{{I}}}^{{{\text{S}}}}$ and $\theta _{{{{II}}}}^{{{\text{S}}}}$ values are observed for type *I* and *II* solution groups, respectively, and JP with a $\theta _{{{{III}}}}^{{{\text{L}}}}$ value is observed for type *III*. The thickness of the midrib was roughly estimated as 300 nm from Fig. 1d, and **p**_V18_ is nearly edge-on (~ 6°).

The orientations of JPs of type *I*–*III* variant pairs were determined experimentally through single trace analysis, as shown in Fig. 1m–o. The observed pairs belonging to type *I*–*III* solution groups were converted to V1/V17, V1/V6, and V1/V16, respectively. The orientations of the traces of JPs for type *I*–*III* correspond to the theoretical ${\mathbf{m}}_{{{I}}}^{{{\text{S}}}}$, ${\mathbf{m}}_{{{{II}}}}^{{{\text{S}}}}$, and ${\mathbf{m}}_{{{{III}}}}^{{{\text{L}}}}$, i.e., (0, 0.707, 0.707), (0.155, 0.699, 0.699), and (100), respectively, in the case of PG (011). The theoretical and average experimental orientations of the JPs are depicted by black and red dots, respectively, in Fig. 1m–o, and are summarized in Supplemental Table S2. The experimental values deviate from theoretical values by less than 4° in the cases of type *I* and *II* variant pairs, and by 7° in the case of type *III* variant pair.

### Observed frequency of variant pairs

We ignore the effect of prior austenite grain boundaries on the variant-pair frequency in the specimens cooled at 77 K and 231 K, although they are considered to be one of the most favorable nucleation sites of variant pairs^48^. There is a sufficient number of variants that do not contact the prior austenite grain boundaries (the number fraction of applicable variants is higher than 85%). A single prior austenite grain does not contain a sufficient number of variants for a variant-pair frequency analysis because 7 of the possible 24 variants are not detected in the specimen cooled at 231 K (Fig. 1d). Therefore, variant maps of 7 prior austenite grains (their NDs are shown in Supplemental Fig. S4) were constructed and used for analysis; we analyzed 568 variant pairs in total. The average volume fraction of residual austenite is ~ 70%. Figure 1p shows the number fractions of variant pairs classified by solution group. The variant-pair frequency, if all the variants are observed and observed equivalently, should be 4% for each of type *I*-*VI*, *IX*, and *X*; 9% for each of type *VII*, *VIII*, and *XI*; and 39% for no solution (NS). The observed frequencies of type *I*–*III* variant pairs greatly exceed 4%, and their cumulative frequency exceeds 70%.

**Figure 2:**
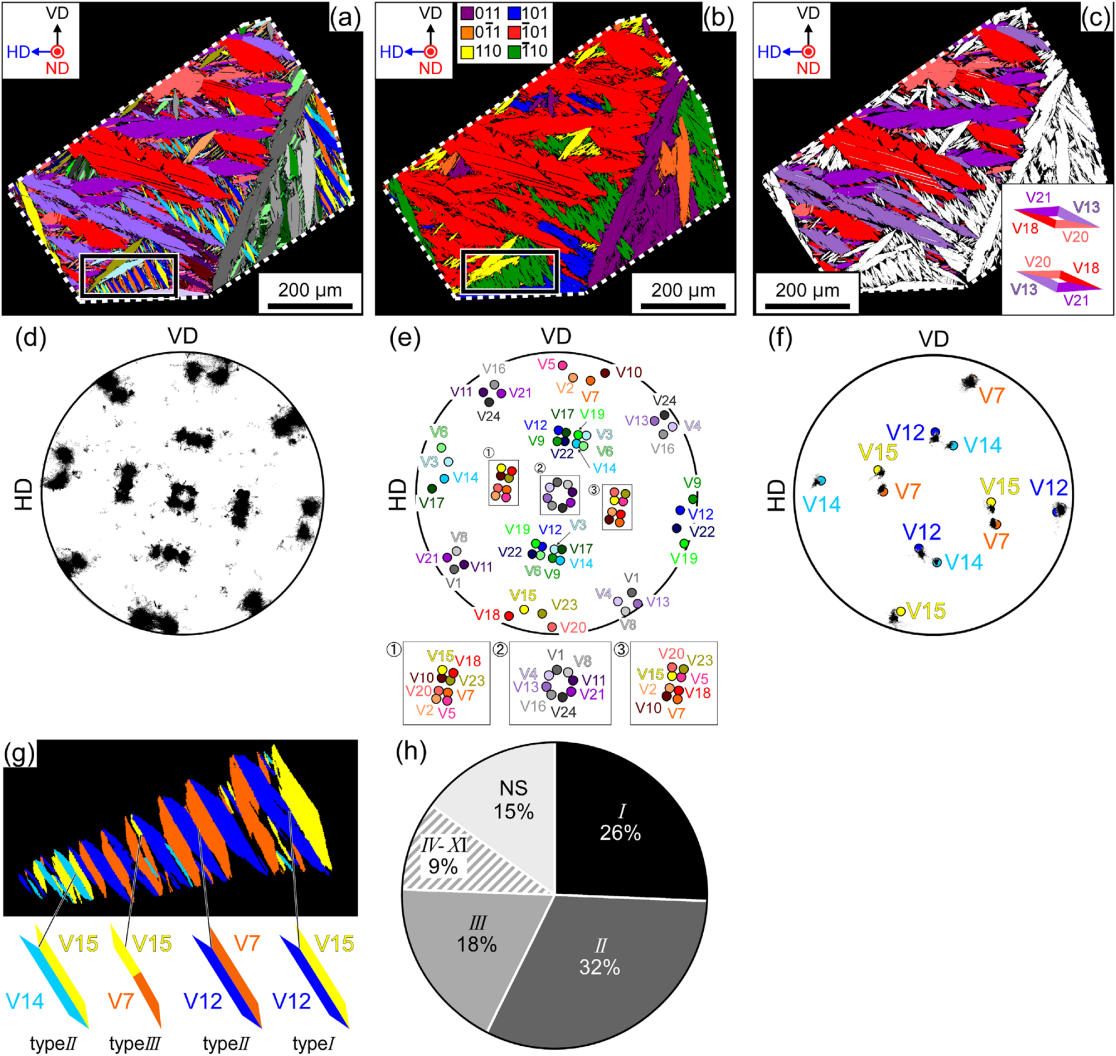
(**a**) Variant map of a specimen cooled at 77 K. The boundary of prior austenite grain is depicted by a dashed line. Black framed region is enlarged in (**g**). The normal direction-vertical direction-horizontal direction (ND-VD-HD) is shown in the inset. (**b**) PG map of the specimen cooled at 77 K. (**c**) Spatial distribution of variants belonging to PG($\bar{1}$01) with lengths exceeding 5 µm. Simulated (001) cross sections of a diamond cluster are shown in the inset. (**d**) 100_ M_ PF of the martensite variants. (**e**) Positions of the 100_ M_ poles of 24 variants when the martensite satisfies the K–S OR. (**f**) 100_ M_ PF for the variants shown in (**g**) and positions of theoretical 100_ M_ poles (K–S OR). (**g**) Variant map of the PG($\bar{1}$10) in the black framed region in (**a**) and (**b**). (**h**) Observed frequency of variant pairs.

Figure 2a displays a variant map and HP traces of the specimen cooled at 77 K (residual austenite volume fraction: 33%). Only one prior austenite grain was analyzed; the ND of the prior austenite grain is [0.015, 0.045, 0.999]. The orientation spread of austenite is 7°, and exceeds that of the austenite in the specimen cooled at 231 K. The measured positions of 100_ M_ poles (Fig. 2d) are consistent with the simulated ones (K–S OR and the IP condition), as shown in Fig. 2e and Supplemental Fig. S6(a). Despite the large orientation spread of austenite, all 24 variants are identified by combining the crystal orientation information and the trace analysis results shown in Fig. S6(b). The difference in the trace angles of two variants with a small misorientation (> 30°) is sufficient for variant identification, which exceeds the orientation spread of austenite.

All 24 variants are detected; however, significant bias exists in their variant number fractions. The most and the least observed variants are V18 (10.5%) and V19 (0.8%), respectively. Figure 2b shows the distribution of variants belonging to the same PG (PG map). Variants belonging to the same PG form clusters, and the number fraction of variants in PG($\bar{1}$01) is the highest, at 36%. This bias implies the existence of 3D spatial inhomogeneity of PG clusters. Figure 2g shows an enlarged view of the area framed in Fig. 2a and b, where variants belong to the PG($\bar{1}$10) cluster. The corresponding 100_ M_ PF, and the positions of the theoretical 100_ M_ poles (K–S OR), are shown in Fig. 2f. Variants form zig-zag patterns and each variant pair is connected by type *I*–*III* JPs (see Supplemental Fig. S3). Figure 2h shows the frequencies of variant pairs; based on the analysis of 4425 pairs, the cumulative frequency of variant pairs belonging to type *I*–*III* solution groups exceeds 70%. This result is consistent with that obtained in a previous study on the variant-pairing tendencies of lenticular martensite^3,28^.

Variant pairs with a small *θ*_*l/k*_ (type *I*, *II*, and *III*) are observed frequently in the specimens cooled at 231 K and 77 K.

## Discussion

Prior to discussing the variant-pair frequency, the formation process of variant pairs and the features of JPs must be clarified. V1 and the other 23 variant pairs are considered as a representative example in the case of K–S notation.

Based on the report that the thickening of lenticular martensite is ~ 3000 times slower than its lengthwise growth, we can estimate that martensite plates grow by ~ 270 nm in the width direction when they grow edge-to-edge on the austenite grain (average grain size: 800 µm)^43^. Because this value is smaller than the width of the midribs in this alloy (300 nm), the thin-plates could probably form a zig–zag pattern first. The fact that the experimentally obtained JPs are consistent with the theoretical ones (Fig. 1m–o) corroborates our assumption.

As for the features of JPs, Fig. 1g–i clearly show that JPs are not {112}_M_ twinning planes. JPs corresponding to type *I*–*III* are incoherent planes because coherent interface formation can occur only in type *V* (V1/V2)^39^ JPs when variants demonstrate K–S OR.

Then, the effects of (1) connection style (acute or obtuse), (2) orientation relationship between the JP of the variant pair and ND of the grain, and (3) area of the JP on the frequency of variant pairs should be analyzed to determine the true variant-pair frequency. Our analysis is based on the data shown in Fig. 2a and three approximations. First, the martensite plates are treated as parallelepipeds comprising HPs; two of the planes are parallel to the JP and other two planes; we focus only on the midrib of the martensite. Second, the HP areas are considered to be much greater than those of the other four planes; the aspect ratio of a lenticular martensite plate is ~ 0.1^49^. Finally, the ND of the austenite, [0.015, 0.045, 0.999], is approximated as [001].

**Figure 3:**
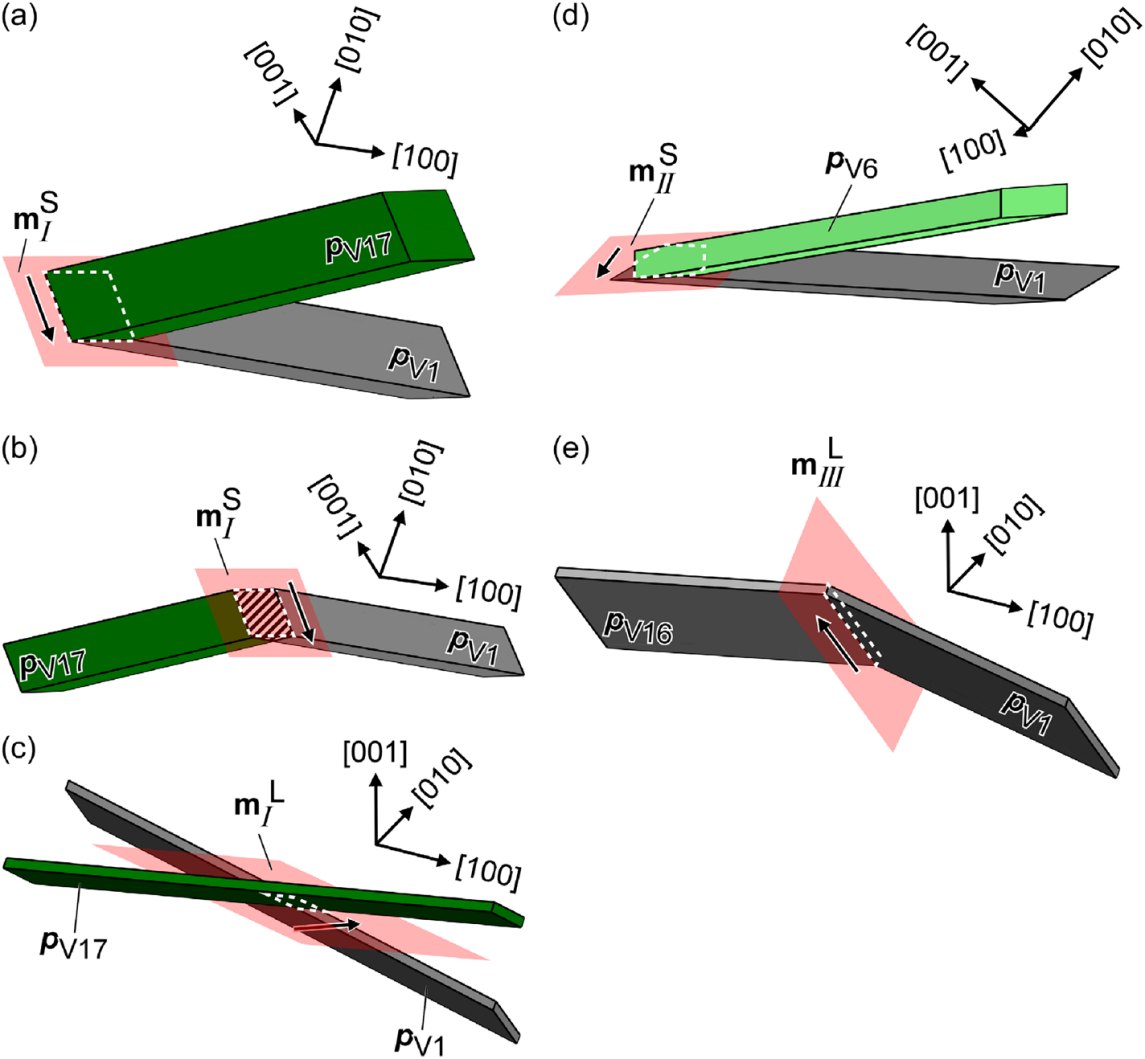
Morphologies of variant pairs: (**a**) acute type *I* with ${\mathbf{m}}_{{{I}}}^{{\text{S}}}$, (**b**) obtuse type *I* with ${\mathbf{m}}_{{{I}}}^{{\text{S}}}$, (**c**) type *I* with ${\mathbf{m}}_{{{I}}}^{{\text{L}}}$, (**d**) acute type *II* with ${\mathbf{m}}_{{{{II}}}}^{{\text{S}}}$, and (**e**) obtuse type *III* with ${\mathbf{m}}_{{{{III}}}}^{{\text{L}}}$. Black arrows indicate the directions of the intersection lines of two HPs.

It is believed that factor (1) affects the priority of variant-pair formation. We consider three or four types of variant-pair morphology in terms of two JPs and connection style, for each solution group, as shown Fig. 3a–c. An acute or obtuse angle is distinguished by the form of variant pair viewed from the direction of the intersection line of two HPs. However, they could not be defined when the intersection line of two HPs is not perpendicular to the normal of JP (discussed later), as shown in Fig. 3c. Figure 3a,b show type *I* variant pairs with the same JP but different connection styles, wherein the formation of the obtuse type *I* variant pair is suppressed because the variant plates interfere in the area hatched in diagonal lines.

Factors (2) and (3) reduce the correlation between the observed 2D and true 3D frequency of variant pairs. In the case of factor (2), variant pairs with edge-on JPs are observed more frequently than variant pairs with face-on JPs because the specimen surface observed must necessarily intersect with the JPs of detected variant pairs. In other words, there is a greater chance that the observed surface intersects with a JP of former than the latter. We can assume that JPs parallel to (001) are rarely observed; in contrast, (100) or (010) JPs are formed by the same type of variant pairs belonging to other PGs. This implies that the effect of factor (2) can be mitigated by considering the observed frequencies of all combinations of variants, as in this study.

In the case of factor (3), the orientation relationship between the JP and HPs of two variants affects the area of the JP. The geometric area of the JP tends to increase when the intersection line of the two HPs is nearly perpendicular to the normal of the JP. When the intersection line of the two HPs is completely perpendicular to the normal of the JP, it is on the JP, and the traces of the two HPs on the JP are parallel (e.g., Fig. 3a,e), maximizing the area of JP. The JPs with normals that are not perpendicular to the intersection lines of the associated HPs are listed in Table 1. There is a possibility that the number of highlighted variant pairs also form preferentially; however, these were not detected. This may be one of the reasons why we did not observe a variant pair with ${\mathbf{m}}_{{{{III}}}}^{{\text{S}}}$.

The risk of JP interference and the orientation relationship between the intersection line of two HPs and JP are summarized in Table 2 for type *I*–*III* variant pairs. The factors that reduce variant-pair frequency (suppressing the formations, while reducing the correlation between the observed 2D and true 3D) are highlighted. JP interference risk is determined by the properties on the left of the table. For type *I* and *II* variant pairs, the preferred morphology is an acute variant pair with $\theta _{{{I}}}^{{{\text{S}}}}$ and $\theta _{{{{II}}}}^{{{\text{S}}}}$, respectively. The obtuse variant pair with $\theta _{{{{III}}}}^{{{\text{L}}}}$ is the preferred morphology of type *III* variant pairs because $\theta _{{{{III}}}}^{{{\text{L}}}}$ is the fourth lowest of the 22 *θ*_*l/k*_ values (Table 1) and there is minimal difference between $\theta _{{{{III}}}}^{{{\text{S}}}}$ and $\theta _{{{{III}}}}^{{{\text{L}}}}$ (0.44°). The morphologies of these variant pairs are depicted in Fig. 3a,d,e.

**Table 2:**
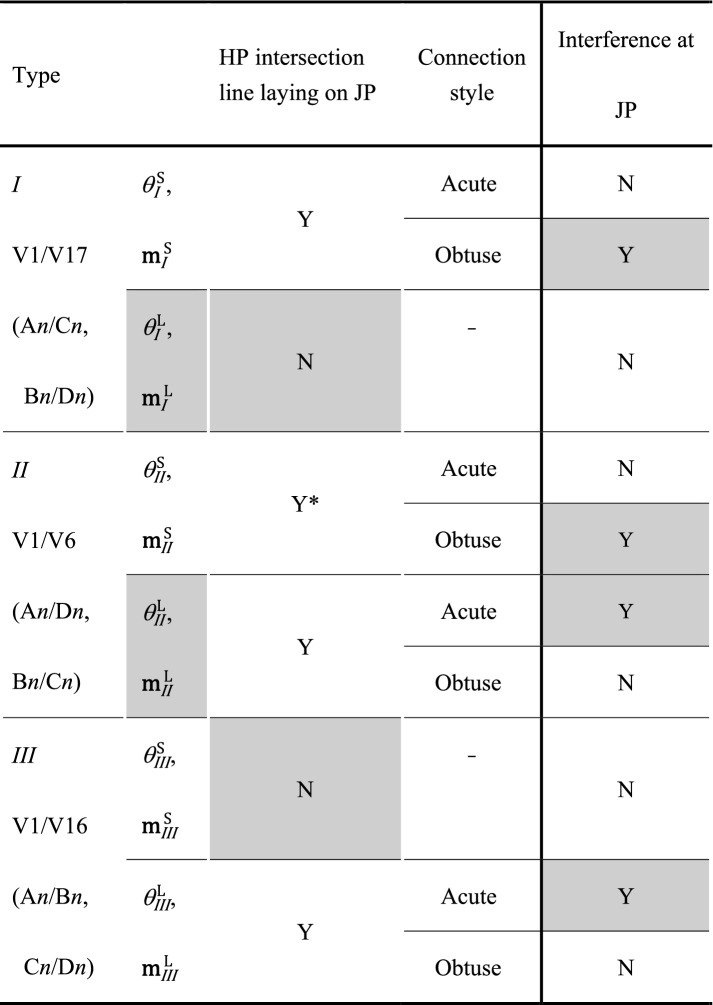
Factors affecting the frequency of type *I–III* variant pairs (variant notation presented by Okamoto is given in parentheses). Factors that reduce the frequency are highlighted.

*Intersection line of the HPs and the normal of the JP has a deviation of 1.2°.

These results imply that the variant-pair frequency evaluated from 2D data does not approximate the true frequency. However, we can conclude that factors (2) and (3) are not the main cause of the high frequencies of the type *I*–*III* variant pairs because other solution groups are not observed despite satisfying the conditions for a high frequency (e.g., the type *IV* variant pair). There is no doubt that the type *I*–*III* variant pairs form preferentially.

The formation of type *I* (V1/V17, A*n*/C*n* and B*n/*D*n*) and type *II* (V1/V6, A*n*/D*n* and B*n/*C*n*) variant pairs is also explained by the accommodation of shape strain^2^. It was considered that variants connect to produce an average shape strain matrix that approaches the identity matrix **I**. The average shape strain matrices for type *I*–*III* variant pairs and PG(011) cluster are shown in Supplemental Table S3. Consistent with the findings of previous studies, Table S3 indicates that a variant cluster comprising four plates belonging to the same PG can accommodate shape strain more effectively than variant pairs^2^. The analysis based on rank-1 connection shows that two variants belonging to the same PG form a JP with a small *θ*_*l/k*_, as shown above. Based on this concept, the preferred morphology of a variant cluster in the Fe-30Ni-0.3C alloy is discussed.

The cumulative degree of incompatibility at JPs should be considered in determining the possibility of variant cluster formation. There is a multitude of variant cluster combinations; thus, we narrowed down candidates based on the following rules: (1) each variant plate connects to two other plates, and (2) the components of variant clusters are acute type *I* and type *II* variant pairs with ${\mathbf{m}}_{{{I}}}^{{{\text{S}}}}$ and ${\mathbf{m}}_{{{{II}}}}^{{{\text{S}}}}$, respectively, and obtuse type *III* variant pairs with ${\mathbf{m}}_{{{{III}}}}^{{{\text{L}}}}$.

**Figure 4:**
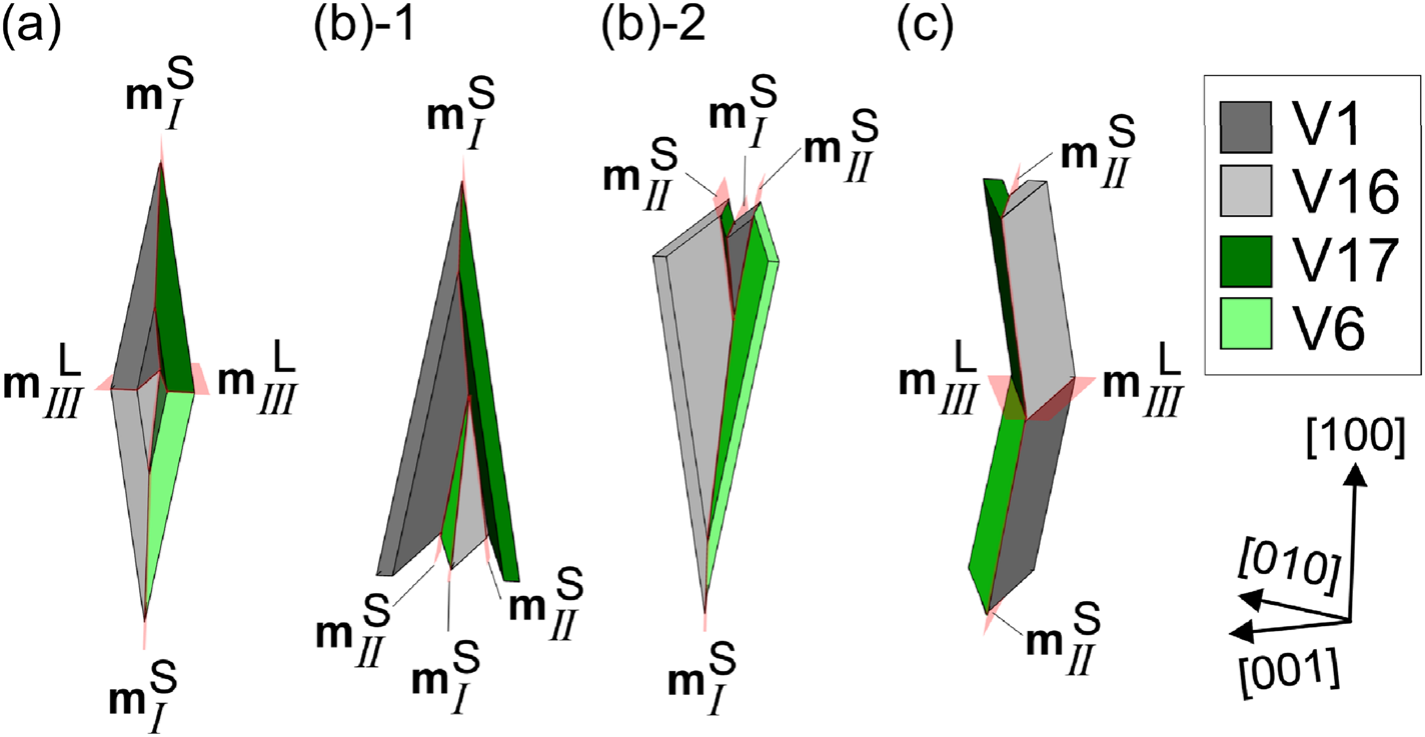
Morphologies of (**a**) diamond, (**b**) composite-spear (CS), and (**c**) composite-kink (CK) clusters. JPs are shown in red.

A diamond cluster comprises two acute type *I* variant pairs with ${\mathbf{m}}_{{{I}}}^{{{\text{S}}}}$ and two obtuse type *III* variant pairs with ${\mathbf{m}}_{{{{III}}}}^{{{\text{L}}}}$, as shown in Fig. 4a. The cumulative rotation at the JPs of a diamond cluster is expressed as follows:

$${\mathbf{Q}}_{{\text{D}}} {\text{ = }}{\mathbf{Q}}_{{{\text{A}n/\text{C}n}}} {\mathbf{Q}}_{{{\text{C}n/\text{D}n}}} {\mathbf{Q}}_{{{\text{D}n/\text{B}n}}} {\mathbf{Q}}_{{{\text{B}n/\text{A}n}}} .$$

In the case of K–S variants belonging to PG(011), it can be rewritten as follows:

$${\mathbf{Q}}_{{\text{D}}} {\text{ = }}{\mathbf{Q}}_{{{\text{V1/V17}}}} {\mathbf{Q}}_{{{\text{V17/V6}}}} {\mathbf{Q}}_{{{\text{V6/V16}}}} {\mathbf{Q}}_{{{\text{V16/V1}}}} .$$

*.*

**Figure 5:**
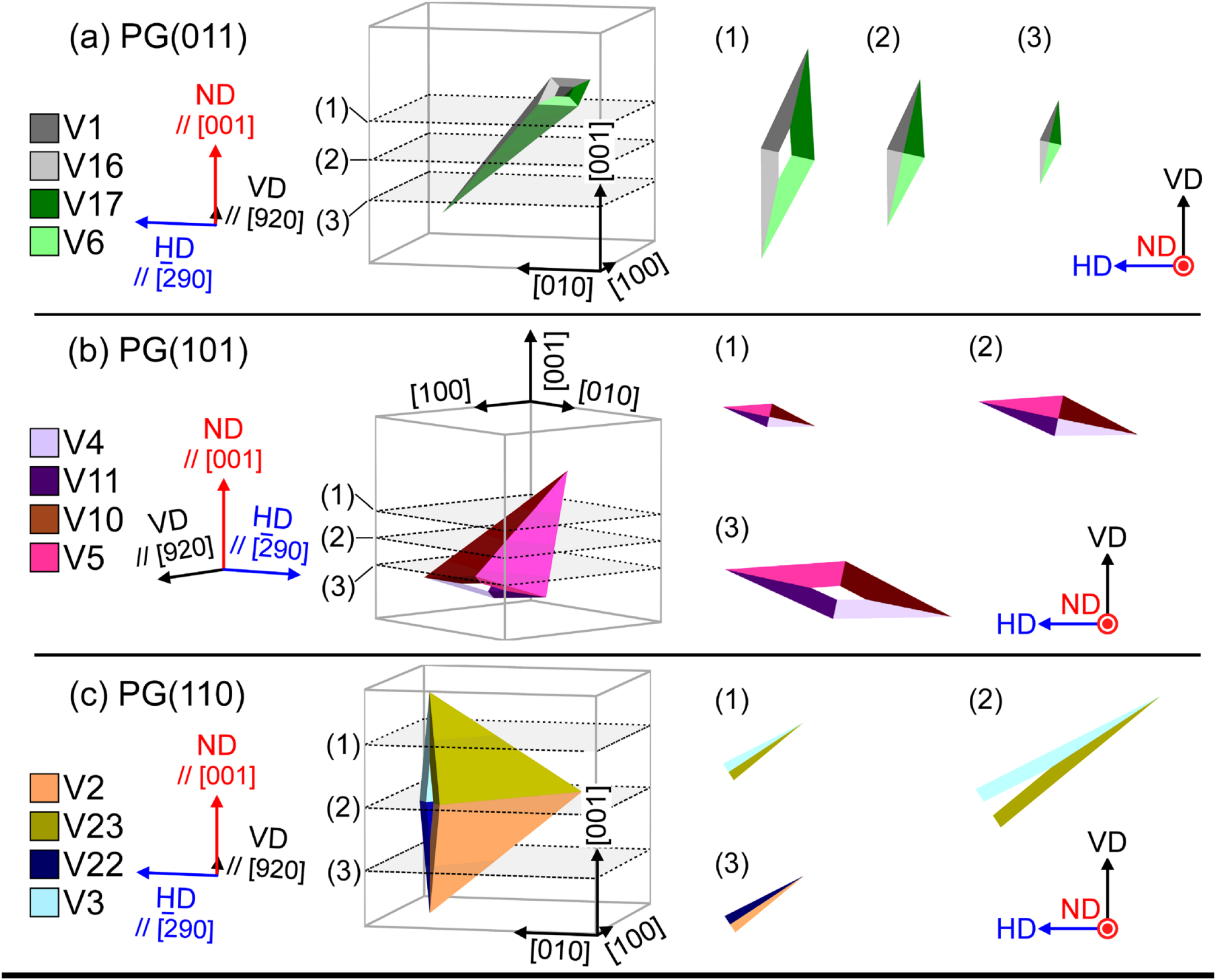
Simulated cross sections of diamond clusters consisting of variants belonging to (**a**) PG(011), (**b**) PG(101), and (**c**) PG(110). The planes surrounded by dashed lines correspond to observed surfaces. The ND-VD-HD coordinate system corresponds to the austenite in Fig. 2a.

**Q**_D_ is rotated through 9.84° to about $\left[ {\overline{{{{0}}{{.005}}}} {{,~0}}{{.707,~}}\overline{{{{0}}{{.707}}}} } \right]$. Therefore, the formation of this cluster is unfavorable in terms of the cumulative degree of incompatibility at the JPs because the magnitude of **Q**_D_ is greater than $\theta _{{{I}}}^{{{\text{S}}}}$ and $\theta _{{{{III}}}}^{{{\text{L}}}}$. Simulated cross sections of diamond clusters comprising variants belonging to PG(011), PG(101), and PG(110) are shown in Fig. 5 (and Supplemental animation 1–3). These three PGs are sufficient for our discussion on ND//[001]. Clusters of variants belonging to PG(0$\bar{1}$1), PG($\bar{1}$01), and PG($\bar{1}$10) have orientation relationships of rotation through 90° or 180° about [001] with PG(011), PG(101), and PG(110), respectively, in terms of the intersection shape of the clusters. A rhombus comprising four variant plates and four traces of JPs always appears in the cross sections of diamond clusters, featuring variants from PG(011), PG(101), PG(0$\bar{1}$1), and PG($\bar{1}$01) obtained through the slicing processes shown in Fig. 5a,b. A V-shape, associated with the type *I* variant pair, always appears in diamond clusters featuring variants from PG(110) and PG($\bar{1}$10), as shown in Fig. 5c. Figure 2c displays the same area as Fig. 2a. Variants belonging to PG($\bar{1}$01), with long axes exceeding 5 µm, are colored; the number fraction of variants belonging to this PG is the highest among the six PGs. The absence of a rhombus in Fig. 2c indicates that the formation of the diamond morphology is suppressed.

**Figure 6:**
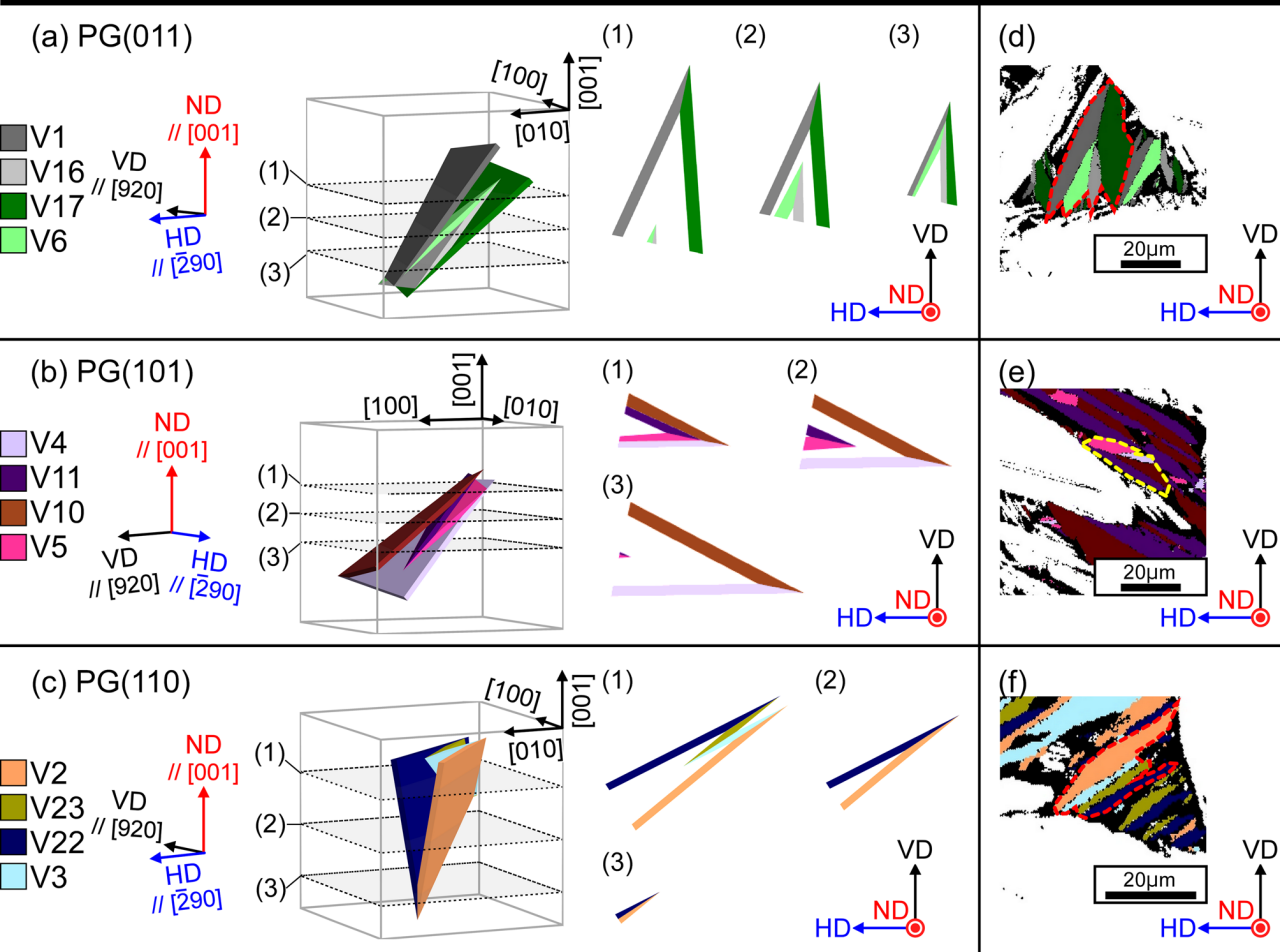
Simulated cross sections of CS clusters featuring variants belonging to (**a**) PG(011), (**b**) PG(101), and (**c**) PG(110). The planes surrounded by dashed lines correspond to observed surfaces. The ND-VD-HD coordinate system corresponds to the austenite in Fig. 2a. Corresponding clusters of four variants from Fig. 2a are shown in (**d**)–(**f**). Note that (**e**) corresponds to CS clusters shown in Fig. 4b–2.

A composite-spear (CS) cluster consists of two acute type *I* variant pairs with ${\mathbf{m}}_{{{I}}}^{{{\text{S}}}}$ and two acute type *II* variant pairs with ${\mathbf{m}}_{{{{II}}}}^{{{\text{S}}}}$, as shown in Fig. 4b–1, b–2, respectively. We can consider two kinds of CS clusters. Figure 6a–c show simulated cross sections of CS clusters, as shown in Fig. 4b–1, featuring variants from PG(011), PG(101), and PG(110), respectively. Assemblies of four variants are obtained under limited slicing conditions for all PGs, as shown in Fig. 6a(3),b(1),c(1) (Supplemental animation 4–6).

The cumulative rotation at the JPs of a CS cluster is as follows:

$${\mathbf{Q}}_{{\text{CS}}} {\text{ = }}{\mathbf{Q}}_{{{\text{A}n/\text{D}n}}} {\mathbf{Q}}_{{{\text{D}n/\text{B}n}}} {\mathbf{Q}}_{{{\text{B}n/\text{C}n}}} {\mathbf{Q}}_{{{\text{C}n/\text{A}n}}} .$$

In the case of K–S variants belonging to PG(011), it can be rewritten as follows:

$${\mathbf{Q}}_{{{\text{CS}}}} {{~ = ~}}{\mathbf{Q}}_{{{\text{V1/V6}}}} {\mathbf{Q}}_{{{\text{V6/V16}}}} {\mathbf{Q}}_{{{\text{V16/V17}}}} {\mathbf{Q}}_{{{\text{V17/V1}}{{.}}}}$$

**Q**_CS_ = **I** (a rotation through 0°). Therefore, the formation of this cluster is favorable in terms of the cumulative degree of incompatibility at the JPs. Further, the normals of all the JPs lie on the (01$\bar{1}$) plane; the orientations of the JPs are (0.155, $\overline{{{{0}}{{.699}}}}$, $\overline{{{{0}}{{.699}}}}$), ($\overline{{{{0}}{{.155}}}}$, $\overline{{{{0}}{{.699}}}}$, $\overline{{{{0}}{{.699}}}}$), and two (011). Hence, the CS clusters meet the three compatibility conditions for the suppression of defects at the quadruple junction point of variants^38^: all the JPs have rank-1 connection solutions, **Q**_CS_ = **I**, and the normals of all the JPs lie on the same plane. These conditions are the same as the formation conditions for a “crossing-twin”^38^. In Fig. 6d–f, PG(011), PG(101), and PG(110) variant clusters that correspond to the simulated cross sections are shown; however, only a few cases are observed.

A composite-kink (CK) cluster comprises two acute type *II* variant pairs with ${\mathbf{m}}_{{{\text{II}}}}^{{{\text{S}}}}$ and two obtuse type *III* variant pairs with ${\mathbf{m}}_{{{\text{III}}}}^{{{\text{L}}}}$, as shown in Fig. 4c. The cumulative rotation at the JPs in a CK cluster is as follows:

$${\mathbf{Q}}_{{\text{CK}}} {\text{ = }}{\mathbf{Q}}_{{{\text{A}n/\text{B}n}}} {\mathbf{Q}}_{{{\text{B}n/\text{C}n}}} {\mathbf{Q}}_{{{\text{C}n/\text{D}n}}} {\mathbf{Q}}_{{{\text{D}n/\text{A}n}}} .$$

In the case of K–S variants belonging to PG(011), it can be rewritten as follows:

$${\mathbf{Q}}_{{{\text{CK}}}} {{~ = ~}}{\mathbf{Q}}_{{{\text{V1/V16}}}} {\mathbf{Q}}_{{{\text{V16/V17}}}} {\mathbf{Q}}_{{{\text{V17/V6}}}} {\mathbf{Q}}_{{{\text{V6/V1}}{{.}}}}$$

The normals of all JPs lie on the (01$\bar{1}$) plane, and **Q**_CK_ = **I** (a rotation through 0°). The orientations of the JPs are (0.155, 0.699, 0.699), (0.155, $\overline{{{{0}}{{.699}}}}$, $\overline{{{{0}}{{.699}}}}$), and two ($\bar{1}$00) in the case of a CK cluster comprising variant pairs belonging to PG(011). CK clusters also meet the above-mentioned compatibility conditions for the suppression of defects at a quadruple junction point of variants^38^. The simulated cross sections of CK clusters shown in Supplemental Fig. S7 show that assemblies of four variants are obtained under limited slicing conditions in cases of variants belonging to PG(011), PG(101), PG(0$\bar{1}$1), and PG($\bar{1}$01). This implies that, similar to CS clusters, CK clusters are rarely observed, and as expected, clusters of four variants belonging to these PGs are not observed experimentally.

The investigated clusters are rarely observed, for different reasons: the cumulative degree of incompatibility at the JPs and narrow window of the slicing condition. However, irrespective of whether type *III* variant pairs with ${\mathbf{m}}_{{{{III}}}}^{{\text{S}}}$ form or not, the quantitative frequencies of variant pairs and clusters determined through 2D analyses are inaccurate. Therefore, 3D microstructural analysis is essential for addressing the limitations associated with 2D analysis approaches.

## Conclusions

The variant-pairing tendencies of lenticular martensite in an Fe–30Ni–0.3C alloy were analyzed based on rank-1 connection, especially at the JPs. The variant pairs formed preferentially were explained successfully in terms of the degree of incompatibility at the JPs (*θ*). The following conclusion were drawn:

1. The *θ* for all 552 possible variant pairs was evaluated, and they form 11 types of JPs. Variants belonging to the same PG form JPs with the first (type *I*: V1/V17, A*n*/C*n* and B*n/*D*n*), second (type *II*: V1/V6, A*n*/D*n* and B*n*/C*n*), and third and fourth smallest *θ* (type *III*: V1/V16, A*n*/B*n* and C*n*/D*n*). Variant pairs with type *I* and *II* JPs are also preferred in terms of self-accommodation.
2. The orientations of JPs were determined by single trace analysis. The deviation between the measured and theoretical orientations of the JPs is less than 4° and 7° for type *I* and *II* and type *III* variant pairs, respectively. For type *III* variant pairs, the observed JP corresponds with the theoretical JP with the larger of two *θ*. Type *I*–*III* variant pairs are observed more frequently than the other variant pairs. The observed cumulative relative frequency of these variant pairs exceeds 70% in the specimens cooled at 77 K and 231 K.
3. The possibility of formation of variant clusters comprising four variants belonging to the same plate group was explored. Diamond clusters composed of two type *I* and two type *III* variant pairs are not formed. The cumulative *θ* of a diamond cluster is greater than the *θ* of the type *III* JP. Thus, the cumulative incompatibility at four JPs suppresses cluster formation. In contrast, the incompatibilities of four JPs cancel each other out in the CS and CK clusters. The former comprises two type *I* and type *II* variant pairs, whereas the latter comprises two type *II* and two type *III* variant pairs. A few CS clusters are observed in the specimen cooled at 77 K.
4. We demonstrated the analytical limitations of 2D approaches used to evaluate the frequency of variant pairs and clusters. It is distinctly possible that the frequency determined from 2D data does not approximate the true frequency because the orientation relationships between JPs and intersection lines between two associated HPs affect the areas of the JPs.

## Methods

### Specimen preparation

An alloy with a nominal composition of Fe-30Ni-0.3C (wt%) was fabricated by Ar-arc melting in an Ar–H_2_ (1%) atmosphere and homogenized at 1273 K for 7.2 ks. A cuboid was cut from the obtained ingot and cold-rolled with a 30% reduction in thickness for every three different planes of cuboid. The cold-rolled cuboid was further homogenized during solution-treatment at 1273 K for 180 ks in an Ar atmosphere, and then quenched in water (average austenite grain size: 800 µm). The chemical composition and martensitic transformation start temperature (*M*s) of the solution-treated specimen are shown in Supplemental Table S4. The former was determined using an inductively coupled plasma-atomic emission spectrometer (ICP-AES, ICPS-8100, SHIMADZU), nitrogen–oxygen analyzer (EMGA-650, HORIBA), and carbon–sulfur analyzer (EMIA-Expert, HORIBA). The latter was determined using a differential scanning calorimeter (DSC, DSC-60 plus, SHIMADZU). Test specimens were sliced from the cuboids and cooled to sub-zero temperatures (231 K (just below the *M*s) and 77 K) for 30 s in a mixture of ethanol and liquid nitrogen to form a martensite microstructure. To prepare the specimens for crystallographic analysis, they were mechanically polished and then electropolished in a mixture of 10% perchloric acid and 90% acetic acid at 273–283 K.

The lattice parameters of the parent and martensite phases were measured by *θ*-2*θ* X-ray diffraction (XRD, X'Pert PRO MPD, PANalytical) at room temperature and corrected using an external standard method. The microstructures were examined by transmission electron microscopy (TEM, JEM-2100, JEOL) and backscattered electron-scanning electron microscopy (BSE-SEM, SU5000, Hitachi). Specimens for TEM were picked up from the electropolished specimen surface using a focused ion beam (FIB, JIB-4500, JEOL). Electron backscatter diffraction (EBSD) analysis was performed using a scanning electron microscope equipped with an EBSD detector (DVC5, TSL Solutions) and an orientation imaging microscopy system (OIM, TSL Solutions) to analyze the crystal orientations. Variants were identified by these orientations and midrib traces regarded as those of HPs. The frequency of variant pairs was evaluated in terms of the number of variant pairs. Therefore, a variant pair formed preferentially if it demonstrated a high frequency.

### Analysis method based on the PTMC

In the case of twinned martensite, based on the geometrically nonlinear theory of martensitic transformation, Eq. (1) can be rewritten as follows:^38^

3 $${\mathbf{Q}}{{'}}{\mathbf{U}}_{{{j}}} ~ - {\mathbf{U}}_{{{i}}} {{ = ~}}{\mathbf{a}}_{{{{1~}}}} \otimes {\mathbf{n}}_{{{1}}} ,$$

4 $${\mathbf{Q}}''{{(}}{\lambda }{\mathbf{QU}}_{{{j}}} {{~ + ~(1 - }}{\lambda }{{)}}{\mathbf{U}}_{{\text{i}}} {{)~ - }}{\mathbf{I}} = {\mathbf{P}}_{{{k}}} {{ - }}{\mathbf{I}} = {\mathbf{d}}_{k} \otimes {\mathbf{p}}_{k} .$$

Equation (3), called the “twinning equation,” describes the rank-1 connection between two Bain correspondence variants *i* and *j* in a martensite plate with deformation gradients ${\mathbf{U}}_{{{i}}}$ and ${\mathbf{U}}_{{{j}}}$; they form a twin as a LID. The twinning shear direction *η*_1_, twin plane normal *K*_1_, magnitude of twinning shear, and rigid rotation correspond to **a**_1_, ${\mathbf{U}}_{{{i}}}^{{{{ - 1}}}} {\mathbf{n}}_{1}$, $\left| {{\mathbf{U}}_{{{i}}}^{{{{ - 1}}}} {\mathbf{n}}_{{{1}}} } \right|\left| {{\mathbf{a}}_{{{1}}} } \right|$, and **Q**′, respectively.

Equation (4) describes the rank-1 connection between austenite and a martensite plate (variant *k*) with shape strain **P**_*k*_. The variables λ, **Q**″, **d**_*k*_, and **p**_*k*_ are the volume fraction of the minor twin, rigid rotation, shape-change vector, and HP normal, respectively, and **I** is an identity matrix. This condition is equivalent to the IP condition at the HP.

In the case of lenticular martensite, midribs consist of thin-plate martensites that are initially formed during martensitic transformation; dislocation is introduced during the growth stage^16^. Thus, we calculated the transformation twin components inside martensite and the IP condition between austenite and midribs before evaluating the incompatibility at JPs based on rank-1 connection. A {112}_M_ twin is adopted as the LID based on the result of Eq. (3). The shape-change vector **d**_*k*_ and HP normal **p**_*k*_ for the 24 possible variants were calculated from Eq. (4).

Based on this information and the crystallography of the JPs between variants evaluated based on rank-1 connection in terms of Eq. (2), the 3D morphology of variant pairs and clusters is discussed; they are depicted using Wolfram Mathematica 12.0.

## Supplementary Information

Supplementary Information.

Supplementary Animation 1.

Supplementary Animation 2.

Supplementary Animation 3.

Supplementary Animation 4.

Supplementary Animation 5.

Supplementary Animation 6.

Supplementary Animation 7.

Supplementary Animation 8.

Supplementary Animation 9.

## Data Availability

All data generated or analyzed during this study are included in this published article (and its Supplementary Information files).
