# Supplementary material for: Analysis of variant-pairing tendencies in lenticular martensite microstructures based on rank-1 connection
Source: Sci Rep. 2021 Jul 22;11:14957. doi: 10.1038/s41598-021-93514-z (PMC8298466; doi:10.1038/s41598-021-93514-z)
Supplement: Supplementary file 1 — Supplementary Information. [file 41598_2021_93514_MOESM1_ESM.pdf]

Supplementary Information

Analysis of variant-pairing tendencies in lenticular martensite microstructures based on rank-1 connection

Yuri Shinohara[a, b*], Satomu Akabane[c], and Tomonari Inamura[a, b]

[a] Laboratory for Materials and Structures, Tokyo Institute of Technology 4259-J3-22, Nagatsuta, Midori-ku, Yokohama 226-8503, Japan

[b] Laboratory for Future Interdisciplinary Research of Science and Technology, Tokyo Institute of Technology 4259-J3-22, Nagatsuta, Midori-ku, Yokohama 226-8503, Japan

[c] Tokyo Institute of Technology 4259-J3-22, Nagatsuta, Midori-ku, Yokohama 226-8503, Japan

[*]Corresponding author

E-mail: shinohara.y.aa@m.titech.ac.jp

Table S1 Notation for this paper.

Eq.	(1)	(2)	(3)	(4)
Two Deformation gradients	**F, G**	$\mathbf{P}_k$, $\mathbf{P}_l$ (shape strain)	$\mathbf{U}_i$, $\mathbf{U}_j$	$\mathbf{P}_k$, **I**
Rigid rotation		$\mathbf{Q}_{l/k}$	**Q'**	**Q''**
Interface normal	**n**	$\mathbf{m}_{l/k}$	$\mathbf{n}_1$	$\mathbf{p}_k$ (HP)
A vector that indicates the discontinuity of deformation gradients	**a**	$\mathbf{b}_{l/k}$	$\mathbf{a}_1$	$\mathbf{d}_k$ (shape-change vector)

$\theta_{l/k}$: magnitude of $\mathbf{Q}_{l/k}$

θ_X^{S} and θ_X^{L}: two $\theta_{l/k}$ of variant pairs belonging to solution group X (smaller and larger).

$\mathbf{m}_X^{\mathrm{S}}$ and $\mathbf{m}_X^{\mathrm{L}}$: two JPs of variant pairs belonging to solution group X (with θ_X^{S} and θ_X^{L}).

λ: volume fraction of minor twin

$\mathbf{Q}_{\mathrm{D}}$, $\mathbf{Q}_{\mathrm{CS}}$ and $\mathbf{Q}_{\mathrm{CK}}$: The cumulative rotation at the JPs of the diamond, CS and CK clusters.

Table S2 Theoretical and experimental JP orientations. The orientation relationships are shown in Fig. 1(j)–(l).

Type	Theoretical	Grains analyzed	Experimental	Deviation
I<br>V1/V17<br>(A1/C1)	$\mathbf{m}_I^{\mathrm{S}}$<br>(011)	Grain 1	$(\overline{0.066}, 0.694, 0.717)$	3.9°
II<br>V1/V6<br>(A1/D1)	$\mathbf{m}_{II}^{\mathrm{S}}$<br>(0.155, 0.699, 0.699)	Grain 1	(0.195, 0.679, 0.708)	2.6°
III<br>V1/V16<br>(A1/B1)	$\mathbf{m}_{III}^{\mathrm{L}}$<br>(100)	Grain 1 and 2	$(0.992, \overline{0.038}, \overline{0.117})$	7.0°

Table S3 Shape strain matrices of an isolated variant plate, variant pairs, and a PG(011) cluster.

Solution group	Average shape strain
Isolated plate<br>V1<br>(A1)	$\begin{pmatrix} 0.992 & -0.035 & -0.028 \\ 0.028 & 1.121 & 0.097 \\ -0.028 & -0.122 & 0.903 \end{pmatrix}$
I<br>V1/V17<br>(A1/C1)	$\begin{pmatrix} 0.992 & -0.003 & 0.003 \\ 0.028 & 1.012 & -0.012 \\ -0.028 & -0.012 & 1.012 \end{pmatrix}$
II<br>V1/V6<br>(A1/D1)	$\begin{pmatrix} 0.992 & -0.031 & -0.031 \\ 0.000 & 1.012 & -0.012 \\ 0.000 & -0.012 & 1.012 \end{pmatrix}$
III<br>V1/V16<br>A1/B1	$\begin{pmatrix} 0.992 & 0 & 0 \\ 0 & 1.121 & 0.097 \\ 0 & -0.122 & 0.903 \end{pmatrix}$
PG(110)	$\begin{pmatrix} 0.992 & 0 & 0 \\ 0 & 1.012 & -0.012 \\ 0 & -0.012 & 1.012 \end{pmatrix}$

Table S4 Chemical composition of the alloy (wt%)

Fe	Ni	C	O	Ti	Cu	*M*s
bal.	29.9	0.28	< 0.003	< 0.7	< 0.002	231 K

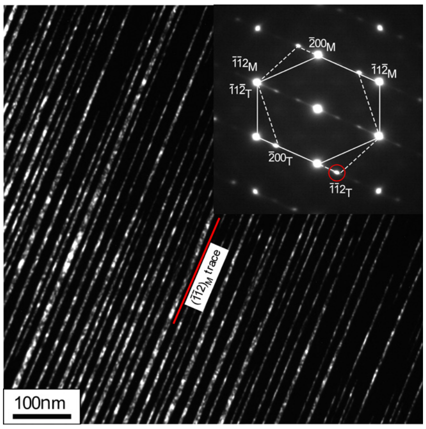

Figure S1 A dark-field image showing the substructure of midrib formed by $\bar{1}\bar{1}2_T$ reflection. Inset shows corresponding diffraction pattern.
Subscript M and T indicate major and minor Bain variants, respectively.

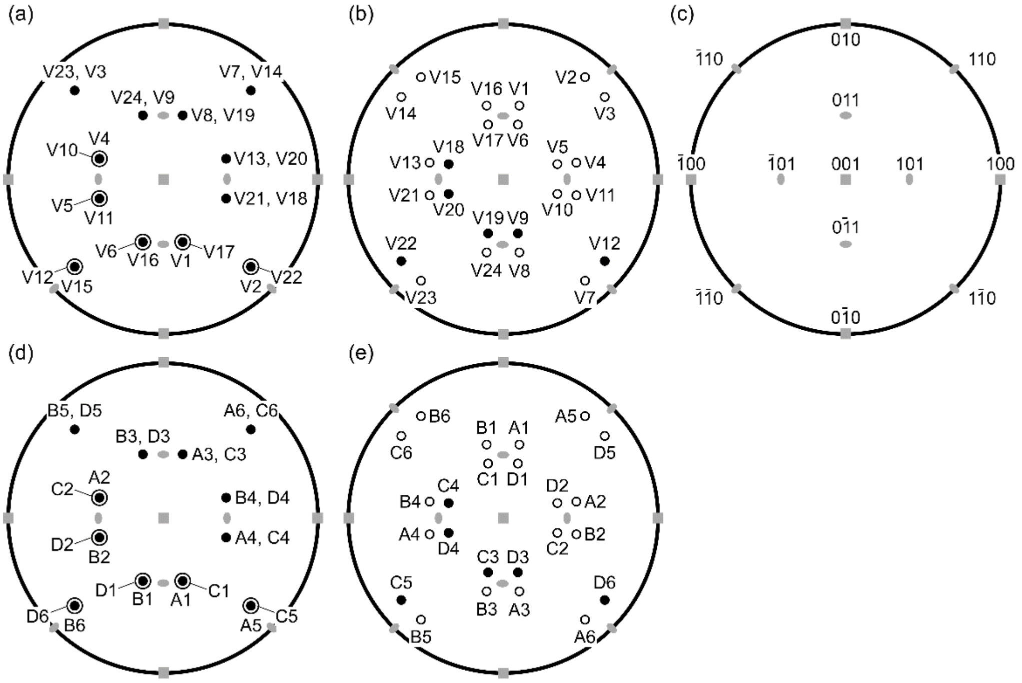

Figure S2 Positions of (a) $\mathbf{d}_k$, and (b) $\mathbf{p}_k$, and a (c) key diagram of the 001 and 110 poles. Positions of (d) $\mathbf{d}_k$, and (e) $\mathbf{p}_k$ labeled using the notation adopted by Okamoto. For $\mathbf{d}_k$ and $\mathbf{p}_k$, poles, symmetry-converted about the origin, are denoted by open symbols.

			PG(011)				PG(101)				PG(0$\bar{1}$1)				PG($\bar{1}$01)				PG(110)				PG($\bar{1}$10)			
			V1	V16	V17	V6	V4	V11	V10	V5	V8	V24	V19	V9	V21	V13	V18	V20	V2	V23	V22	V3	V7	V15	V14	V12
			A1	B1	C1	D1	A2	B2	C2	D2	A3	B3	C3	D3	A4	B4	C4	D4	A5	B5	C5	D5	A6	B6	C6	D6
PG(011)	V1	A1		III	I	II	VI	VIII	NS	NS	NS	X	XI	XI	IV	VIII	NS	NS	V	VII	NS	NS	IX	VII	NS	NS
	V16	B1	III		II	I	VIII	IV	NS	NS	X	NS	XI	XI	VIII	VI	NS	NS	VII	IX	NS	NS	VII	V	NS	NS
	V17	C1	I	II		III	NS	NS	IX	VII	XI	XI	X	NS	NS	NS	V	VII	NS	NS	IV	VIII	NS	NS	VI	VIII
	V6	D1	II	I	III		NS	NS	VII	V	XI	XI	NS	X	NS	NS	VII	IX	NS	NS	VIII	VI	NS	NS	VIII	IV
PG(101)	V4	A2	VI	VIII	NS	NS		III	I	II	VIII	IV	NS	NS	X	NS	XI	XI	NS	NS	VII	V	NS	NS	IX	VII
	V11	B2	VIII	IV	NS	NS	III		II	I	VI	VIII	NS	NS	NS	X	XI	XI	NS	NS	IX	VII	NS	NS	VII	V
	V10	C2	NS	NS	IX	VII	I	II		III	NS	NS	VII	V	XI	XI	NS	X	VIII	IV	NS	NS	VI	VIII	NS	NS
	V5	D2	NS	NS	VII	V	II	I	III		NS	NS	IX	VII	XI	XI	X	NS	VI	VIII	NS	NS	VIII	IV	NS	NS
PG(0$\bar{1}$1)	V8	A3	NS	X	XI	XI	VIII	VI	NS	NS		III	I	II	VIII	IV	NS	NS	IX	VII	NS	NS	V	VII	NS	NS
	V24	B3	X	NS	XI	XI	IV	VIII	NS	NS	III		II	I	VI	VIII	NS	NS	VII	V	NS	NS	VII	IX	NS	NS
	V19	C3	XI	XI	X	NS	NS	NS	VII	IX	I	II		III	NS	NS	VII	V	NS	NS	VI	VIII	NS	NS	IV	VIII
	V9	D3	XI	XI	NS	X	NS	NS	V	VII	II	I	III		NS	NS	IX	VII	NS	NS	VIII	IV	NS	NS	VIII	VI
PG($\bar{1}$01)	V21	A4	IV	VIII	NS	NS	X	NS	XI	XI	VIII	VI	NS	NS		III	I	II	NS	NS	V	VII	NS	NS	VII	IX
	V13	B4	VIII	VI	NS	NS	NS	X	XI	XI	IV	VIII	NS	NS	III		II	I	NS	NS	VII	IX	NS	NS	V	VII
	V18	C4	NS	NS	V	VII	XI	XI	NS	X	NS	NS	VII	IX	I	II		III	IV	VIII	NS	NS	VIII	VI	NS	NS
	V20	D4	NS	NS	VII	IX	XI	XI	X	NS	NS	NS	V	VII	II	I	III		VIII	VI	NS	NS	IV	VIII	NS	NS
PG(110)	V2	A5	V	VII	NS	NS	NS	NS	VIII	VI	IX	VII	NS	NS	NS	NS	IV	VIII		III	I	II	NS	X	XI	XI
	V23	B5	VII	IX	NS	NS	NS	NS	IV	VIII	VII	V	NS	NS	NS	NS	VIII	VI	III		II	I	X	NS	XI	XI
	V22	C5	NS	NS	IV	VIII	VII	IX	NS	NS	NS	NS	VI	VIII	V	VII	NS	NS	I	II		III	XI	XI	X	NS
	V3	D5	NS	NS	VIII	VI	V	VII	NS	NS	NS	NS	VIII	IV	VII	IX	NS	NS	II	I	III		XI	XI	NS	X
PG($\bar{1}$10)	V7	A6	IX	VII	NS	NS	NS	NS	VI	VIII	V	VII	NS	NS	NS	NS	VIII	IV	NS	X	XI	XI		III	I	II
	V15	B6	VII	V	NS	NS	NS	NS	VIII	IV	VII	IX	NS	NS	NS	NS	VI	VIII	X	NS	XI	XI	III		II	I
	V14	C6	NS	NS	VI	VIII	IX	VII	NS	NS	NS	NS	IV	VIII	VII	V	NS	NS	XI	XI	X	NS	I	II		III
	V12	D6	NS	NS	VIII	IV	VII	V	NS	NS	NS	NS	VIII	VI	IX	VII	NS	NS	XI	XI	NS	X	II	I	III	

Figure S3 Solution groups of each variant pair. The definitions of the six PGs are also shown.

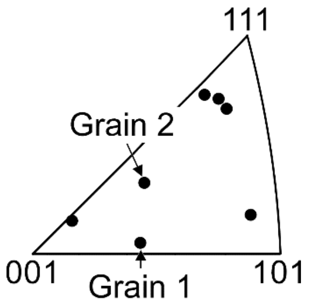

Figure S4 Grain orientation of prior austenite used for analysis of the specimen cooled at just below the *M*s.

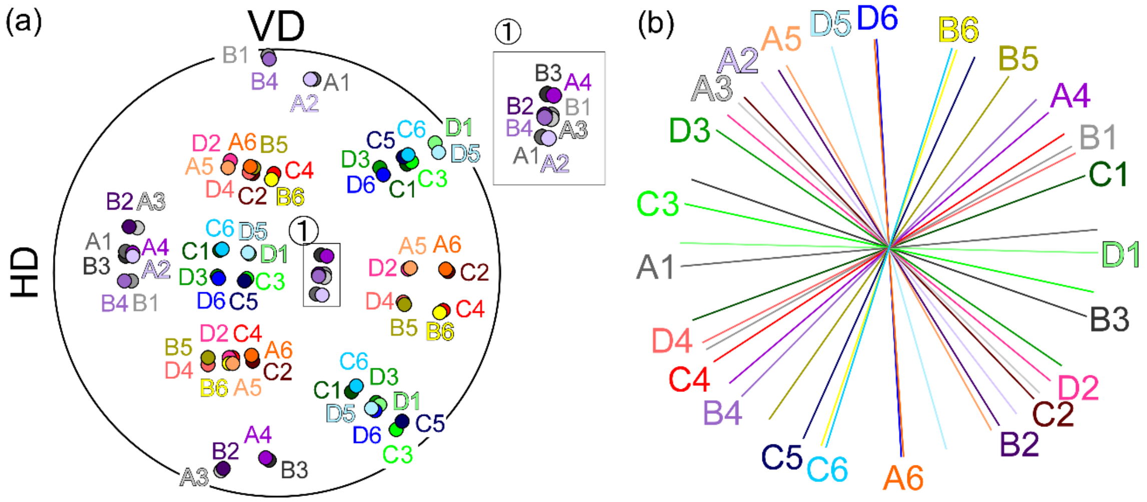

Figure S5 (a) Positions of the 100_M poles of 24 variants of a specimen cooled at 231 K when the martensite satisfies the IP condition. (b) The HP traces in the midrib calculated from the IP condition.

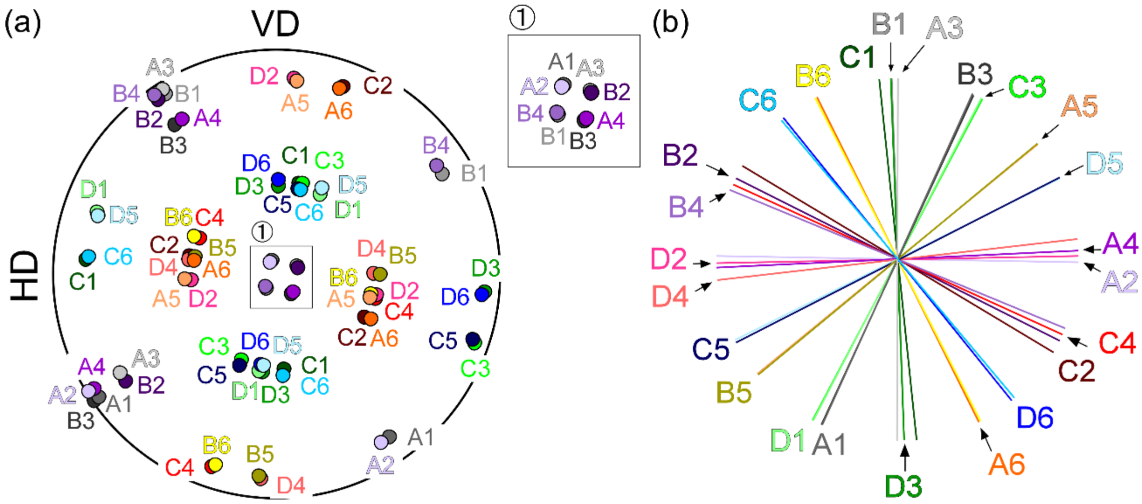

Figure S6 (a) Positions of the 100_M poles of 24 variants of a specimen cooled at 77 K when the martensite satisfies the IP condition. (b) The HP traces in the midrib calculated from the IP condition.

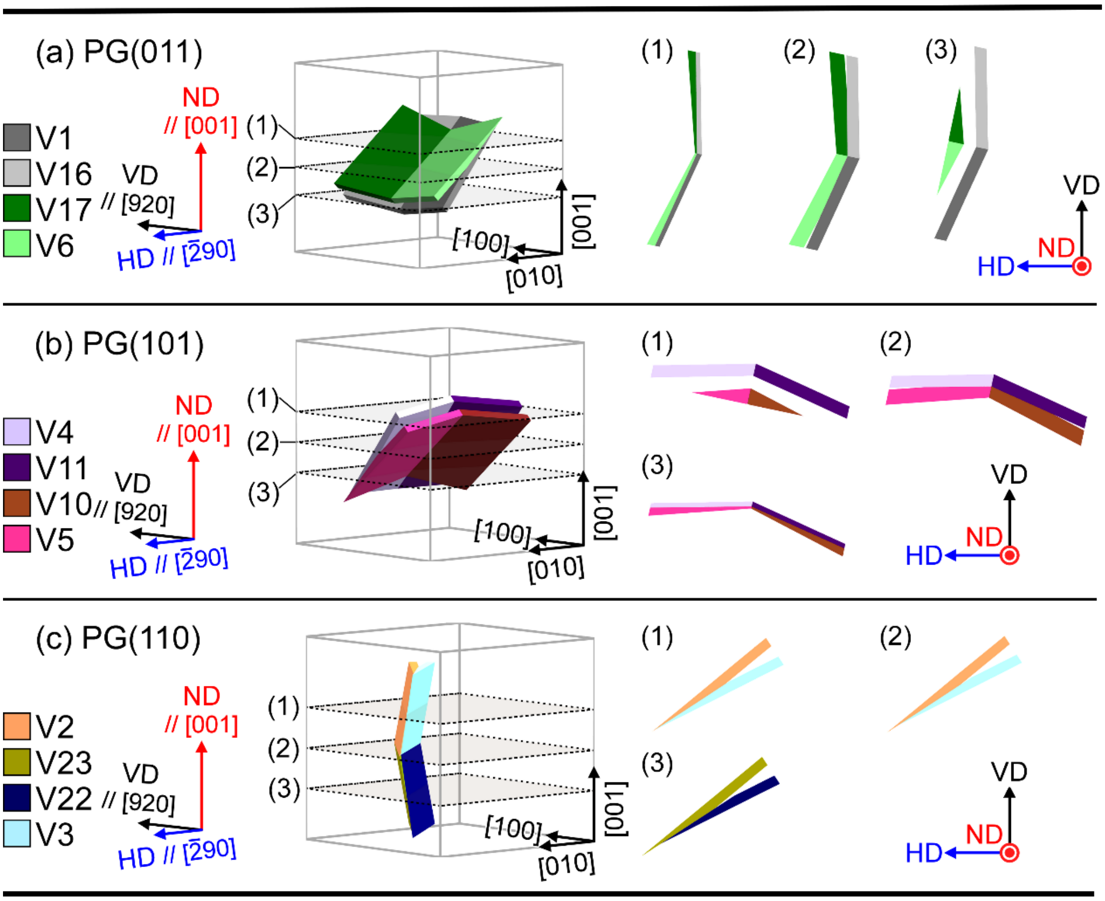

Figure S7 Simulated cross sections of CK clusters featuring variants belonging to (a) PG(011), (b) PG(101), and (c) PG(110). The planes surrounded by dashed lines correspond to observed surfaces. The ND-VD-HD coordinate system corresponds to the austenite in Fig. 2(a).

Supplemental animation

SA1 (**a**) Morphology and (**b**) simulated cross section of a diamond cluster consisting of variants belonging to PG(011). The planes in gray with dashed lines correspond to observed surfaces. The ND-VD-HD coordinate system corresponds to the austenite in Fig. 2(a).

SA2 (**a**) Morphology and (**b**) simulated cross section of a diamond cluster consisting of variants belonging to PG(101). The planes in gray with dashed lines correspond to observed surfaces. The ND-VD-HD coordinate system corresponds to the austenite in Fig. 2(a).

SA3 (**a**) Morphology and (**b**) simulated cross section of a diamond cluster consisting of variants belonging to PG(110). The planes in gray with dashed lines correspond to observed surfaces. The ND-VD-HD coordinate system corresponds to the austenite in Fig. 2(a).

SA4 (**a**) Morphology and (**b**) simulated cross section of a CS cluster featuring variants belonging to PG(011). The planes in gray with dashed lines correspond to observed surfaces. The ND-VD-HD coordinate system corresponds to the austenite in Fig. 2(a).

SA5 (**a**) Morphology and (**b**) simulated cross section of a CS cluster featuring variants belonging to PG(101). The planes in gray with dashed lines correspond to observed surfaces. The ND-VD-HD coordinate system corresponds to the austenite in Fig. 2(a).

SA6 (**a**) Morphology and (**b**) simulated cross section of a CS cluster featuring variants belonging to PG(110). The planes in gray with dashed lines correspond to observed surfaces. The ND-VD-HD coordinate system corresponds to the austenite in Fig. 2(a).

SA7 (**a**) Morphology and (**b**) simulated cross section of a CK cluster featuring variants belonging to PG(011). The planes in gray with dashed lines correspond to observed surfaces. The ND-VD-HD coordinate system corresponds to the austenite in Fig. 2(a).

SA8 (**a**) Morphology and (**b**) simulated cross section of a CK cluster featuring variants belonging to PG(101). The planes in gray with dashed lines correspond to observed surfaces. The ND-VD-HD coordinate system corresponds to the austenite in Fig. 2(a).

SA9 (**a**) Morphology and (**b**) simulated cross section of a CK cluster featuring variants belonging to PG(110). The planes in gray with dashed lines correspond to observed surfaces. The ND-VD-HD coordinate system corresponds to the austenite in Fig. 2(a).
