# Supplementary figures and images for: Analysis of variant-pairing tendencies in lenticular martensite microstructures based on rank-1 connection
Source: Sci Rep. 2021 Jul 22;11:14957. doi: 10.1038/s41598-021-93514-z (PMC8298466; doi:10.1038/s41598-021-93514-z)

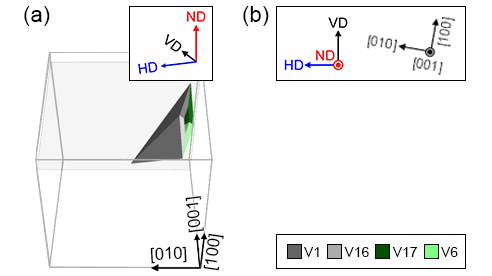

Supplement: Supplementary file 2 — Supplementary Animation 1. [file 41598_2021_93514_MOESM2_ESM.gif]

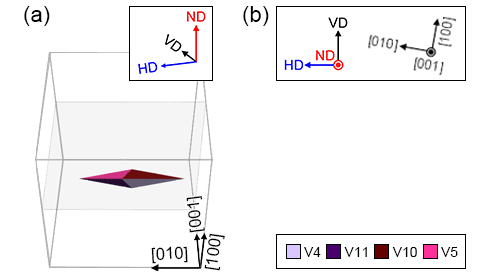

Supplement: Supplementary file 3 — Supplementary Animation 2. [file 41598_2021_93514_MOESM3_ESM.gif]

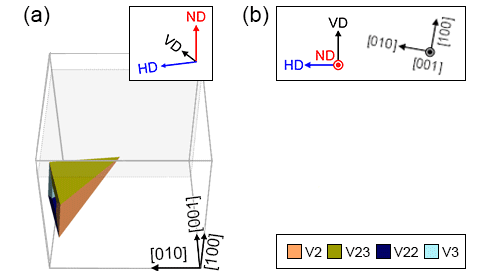

Supplement: Supplementary file 4 — Supplementary Animation 3. [file 41598_2021_93514_MOESM4_ESM.gif]

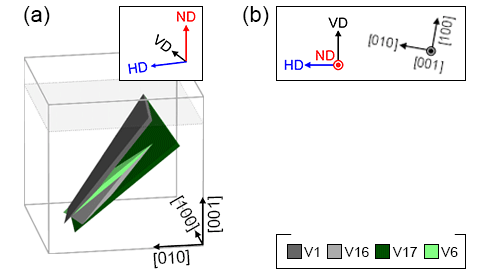

Supplement: Supplementary file 5 — Supplementary Animation 4. [file 41598_2021_93514_MOESM5_ESM.gif]

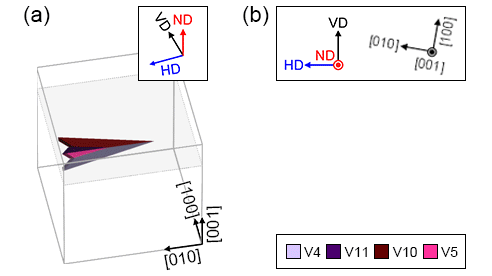

Supplement: Supplementary file 6 — Supplementary Animation 5. [file 41598_2021_93514_MOESM6_ESM.gif]

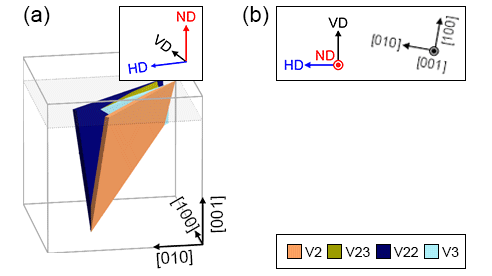

Supplement: Supplementary file 7 — Supplementary Animation 6. [file 41598_2021_93514_MOESM7_ESM.gif]

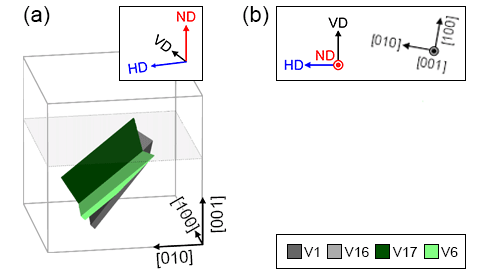

Supplement: Supplementary file 8 — Supplementary Animation 7. [file 41598_2021_93514_MOESM8_ESM.gif]

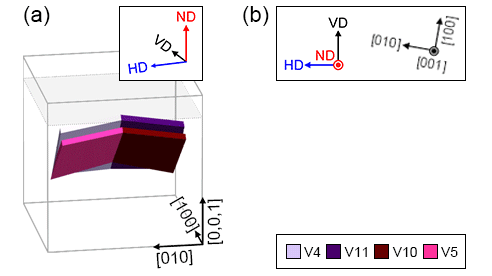

Supplement: Supplementary file 9 — Supplementary Animation 8. [file 41598_2021_93514_MOESM9_ESM.gif]

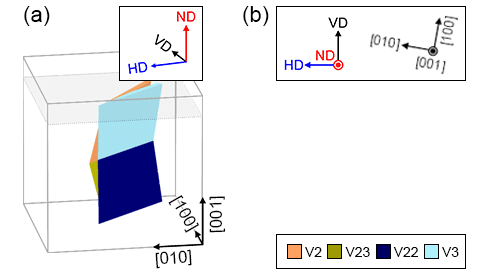

Supplement: Supplementary file 10 — Supplementary Animation 9. [file 41598_2021_93514_MOESM10_ESM.gif]
